# Imposing calculations: The visibility and invisibility of harm in the Mackenzie Gas Project environmental assessment

**DOI:** 10.3389/fsoc.2022.1056277

**Published:** 2023-01-13

**Authors:** Carly Dokis

**Affiliations:** Department of Anthropology, Nipissing University, North Bay, ON, Canada

**Keywords:** extractive industries, pipelines, environmental impact assessment, Mackenzie Gas Project, Dene, Northwest Territories, indigenous participation in resource management

## Abstract

Environmental assessment is an institutional apparatus through which proponents concede harm associated with extractive projects. Within these processes proponents define the nature and scope of harm, which is made visible through the production of indicators and measurements and made manageable through mitigation measures or economic compensation. That the activities of extractive industries may have effects on surrounding ecologies is rarely in question; proponents of extractive projects regularly concede that their activities will result in negative (but also positive) changes to environments and communities. What is often contested in the course of environmental assessment and regulatory processes is the “significance” of the impacts identified, the nature of the harm caused, and whether or not it is possible or acceptable to accommodate it. Drawing from ethnographic fieldwork conducted in the Sahtu Settlement Area, NWT during the Mackenzie Gas Project environmental assessment, along with regulatory documents and transcripts, this paper examines how proponents and regulatory regimes work to make the impacts of extractive industries visible, and how these logics deviate discursively and materially from many Indigenous peoples' understandings of appropriate relationships between human beings and nature.

## 1. Introduction

*In the event of an oil spill there could be damage to the water, to the land, to our wildlife, to our fish. “Might,” it says. I think that maybe that's a typo. The statement that is written says it—it says it might, but I don't think that's correct. It should be written—that typo should be taken out and it should be written “will,” because that's what will happen in the event of a spill*.

*Michael Neyelle, Community Hearings for the Joint Review Panel for the Mackenzie Gas Project, Déline NWT*.

In the 1970s, the Mackenzie Valley Pipeline Inquiry helped to establish a “new social context” for northern development including advocating for the negotiation of comprehensive land claim agreements and the creation of regulatory structures that facilitate Indigenous peoples' inclusion in decision-making surrounding extractive projects (Kulchyski and Bernauer, 2014). Since then, there have been substantial changes in land management regimes in the Northwest Territories of Canada. These new regulatory and environmental assessment institutions were born out of provisions of comprehensive land claims settled with the Inuvialuit, Gwich'in and Sahtu Dene and Metis peoples and sought to formalize Indigenous peoples' participation in land management decisions, including their representation on regulatory boards and involvement in public hearings.

High prices for oil and gas and other commodities in the early 21st century led to a number of proposed extractive mega-projects and associated infrastructures such as BHP's Ekati Diamond Mine, the proposed Mackenzie Gas Project, and others, that fell under the jurisdiction of these emerging regulatory regimes. While the emergence of these regimes pre-date the *2030 Agenda for Sustainable Development* adopted by the United Nations in 2015, they share many of the same goals including recognizing the need to consider the interconnected nature of human, cultural, and environmental impacts as a part of sustainable development, and to build participatory and representative institutions to facilitate this process. The sixteenth SDG specifically addresses the need to “promote peaceful and inclusive societies for sustainable development, provide access to justice for all and build effective, accountable and inclusive institutions.” *Canada's 2030 Agenda National Strategy* likewise recognizes the importance of inclusivity, accountability and transparency, and the unique importance of the governments' commitment to reconciliation with Indigenous peoples by including Indigenous peoples and organizations in meaningful ways in decision-making (SDGU, 2019).

Yet, as studies have shown, the development of new participatory regulatory institutions does not automatically translate into more representative or inclusive decision-making processes, especially when they continue to be modeled upon Euro-centric institutional and conceptual frameworks (Stevenson, 1996; White, 2006; Westman, 2013a; Coulthard, 2014; Dokis, 2015; Baker and Westman, 2018; Joly et al., 2018). In the Northwest Territories post-land claim era, new regulatory institutions were established and were tasked not only with assessing proposed large-scale extractive projects, but also with how to assemble and represent various publics in the environmental assessment process, a process already ripe with conceptual trouble. As Andrew Barry has pointed out, publics are increasingly constituted as collectives that are called upon to mobilize in relation to a problem, issue, or an object, and as such we should attend to the diverse techniques and technologies employed to assemble them and to speak on their behalf. As Barry (2013, p. 97) notes, following Althusser, publics are often ‘hailed' into already existing ideological and institutional apparatuses that have a continuing presence, and often have “particular forms of speech, employ specialist forms of expertise and technical devices, and may involve well-developed procedures as to how they should be used”. In the context of the Northwest Territories, while the *Mackenzie Valley Resource Management Act* was transformative in that it formalized the inclusion of Indigenous peoples' views and concerns about the impacts of extractive projects, it did not remake the regulatory process anew. Rather, many of the ideological, ontological, and axiological underpinnings of the environmental assessment process, as well as the particular spaces and procedures used to elicit input from various publics, reveal deep entanglements in what Willow (2016, p. 2) has described as extractivst logics that work to “pave the way for extractive industries success”. Consequently, these processes are intimately caught up in the continuation of settler colonialism and associated material and ontological dispossession of Indigenous peoples.

The objectives of this paper are to tease out some of these logics through an examination of how particular narratives of harm were produced and contained within environmental assessment documents and hearings for the proposed Mackenzie Gas Project. I explore how proponents, through environmental assessment documents and public statements, work to make the impacts of extractive industries visible, and how these logics deviate from many Indigenous peoples' understandings of caretaking relations (TallBear, 2019, p. 25). The nature of harms described in environmental assessments do not exist as innate elements of ecological or social disruptions, but rather are sociomaterial constructions that are open-ended and uncertain (Westman, 2013a; Sawyer, 2015), leaving room for multiple interpretations and renderings that draw on diverse understandings and experiences of the world. A significant problem for environmental assessment processes, like the one associated with the Mackenzie Valley pipeline, is how to deal with multiple and contested interpretations of the potential impacts of extractive projects.

I draw on an analysis of documents submitted by Imperial Oil for the environmental assessment of the Mackenzie Gas Project, along with attendance at regulatory hearings held throughout the Sahtu region of the Northwest Territories and fieldwork carried out in three Sahtu Dene communities from 2004 to 2008. I argue that the narratives of harm produced by proponents work to make the environmental harms associated with the pipeline and related infrastructure visible and calculable for regulatory authorities in particular ways that highlight Eurocentric hierarchies of life and enactments of property relations. However, the manifestation of these quantitative representations in environmental assessment documents and hearings simultaneously subverts other understandings of the significance of these impacts as Sahtu Dene conceptions of harm are often rendered invisible precisely because they resist such quantification and commodification.

## 2. Recognizing harm in environmental assessment

Environmental assessment has been an important avenue for identifying and understanding the ecological impacts of extractive industries and infrastructure projects and has been mandated in Canada since 1972 (Darling et al., 2018). Wright et al. (2013, p. 72) identifies that the intent of environmental assessments is, “to guide environmentally responsible management practice through an impartial, objective, scientifically-based, thorough, comprehensive and up-to-date description and discussion of: the baseline environment; current, planned and potential human activities; and expected impacts of these activities individually and cumulatively”. Yet, Wright et al. (2013) also note that environmental assessments often fall short of these goals due to a variety of factors. Some of these factors are conceptual, such as the inherent uncertainty of the full extent of the impacts of extractive industries and ill-defined concepts or regulations (Franks et al., 2013). Other factors involve practical issues such as restricted budgets or rushed timelines (Franks et al., 2010; Udofia et al., 2017), and potential biases that may arise when environmental impact statements are conducted or contracted by proponents of extractive projects (Westman, 2013a; Baker and Westman, 2018; Larson, 2018; Arsenault et al., 2019).

In Canada, environmental assessment involves institutional apparatuses through which proponents concede harm associated with extractive projects through the submission of their environmental impact statement. Within these processes, proponents attempt to define the nature and scope of harm, which is made legible through the production of quantifiable indicators and measurements, and made manageable through technological intervention, or by providing economic compensation. That the activities of extractive industries may have effects on surrounding ecologies and communities is rarely in question, proponents of extractive projects regularly concede that their activities will result in negative (but also positive) changes to environments and communities. What is often contested in the course of environmental assessment and regulatory processes is the “significance” of the impacts identified; the nature of the harm caused, and whether or not it is possible or acceptable to accommodate it.

Literature on environmental assessment in the Canadian North suggests that proponents frequently predict that their activities will have “no significant impact” on the environment or communities who depend on surrounding ecologies for their livelihoods (Westman, 2013a; Collard et al., 2020). These claims are made even in instances when extractive processes leave undeniable ecological footprints, such as in the oil sands regions in northern Alberta, or when surrounding Indigenous communities' express concerns about their inability to exercise Aboriginal or Treaty rights to hunt, fish, or gather medicines on lands taken up for extractive purposes (Baker and Westman, 2018; Lewis et al., 2020). Indigenous peoples across the Canadian North have experienced adverse impacts of various extractivist frontiers on surrounding ecologies, traditional land use, community wellness, social cohesion, cultural continuity, and spirituality (Kirmayer et al., 2000; Parlee et al., 2018). Although the scope and intensity of both direct and in-direct impacts of any given project are context-specific, a number of studies suggest that the impacts of extractive industries are experienced by Indigenous peoples as a form of environmental injustice (Keeling and Sandlos, 2009; Horowitz et al., 2018) and displacement (Jackson, 2011) where anxieties and concerns about contaminants and altered lifeways coexist with deep attachments to landscapes that have sustained communities for generations. As Lewis et al. (2020, p. 68) write, “this is a story that has been told many times by many Indigenous communities, yet there is a continued failure to grasp the reality of how land displacement and environmental dispossession negatively impacts Indigenous people”.

Proponents of extractive projects seek to manage harms associated with their activities in a number of ways including occasionally re-routing activities or modifying project plans, implementing policies for workers and personnel, and introducing technological interventions intended to reduce ecological destruction. When ecological destruction is unavoidable, as it is in most extractive projects, proponents frequently claim that the destruction can be partially or fully ameliorated by processes of remediation or restoration. Within environmental impact statements, ecologies that are adversely impacted by extractive industries are often presented as being improved after technological restoration. Clinton Westman, for example, has documented how impacts on hunting, fishing, and plant gathering in the environmental impact assessment for Syncrude's Aurora mine were represented as short term (during the construction and operation phase), and that access to these activities would be improved following oil sands development and reclamation. Westman (2013a, p. 139) notes that “these forecasts do not acknowledge the feeling held by many active foragers that one cannot effectively gather efficacious medicinal plants on land that has been used as a mine, given the damage that has been done to the spirit of the place”. This technological framing of the impacts of extractive industries works to depoliticize the intimate connections between capitalism, settler colonialism, and Indigenous dispossession, and conceals wider political ecologies that contribute to environmental injustice both within Canada and more globally (Keeling and Sandlos, 2009; McCreary, 2014; McGregor, 2015; Zalik, 2015; Fernández-Llamazares et al., 2019).

When remediation of harm is not possible proponents often claim that the impacts caused by extractive industries can be mitigated through economic compensation. Impact Benefits Agreements (IBAs), for example, are required as part of land-claim mandated environmental assessment processes in the Northwest Territories. These formal agreements reached between corporations and Indigenous communities help to secure particular benefits stemming from extractive industries, compensate for social and economic disruption, provide employment opportunities for Indigenous community members and development opportunities for Indigenous-owned businesses, fund the improvement of community infrastructure, and occasionally provide funding for cultural activities (Kennett, 1999; O'Faircheallaigh, 1999; Galbraith et al., 2007). Yet, they are also a means of monetizing the impacts of extractive industries, and of interpolating Indigenous peoples into wage economies while simultaneously undermining subsistence livelihoods (Garvie and Shaw, 2015; St-Laurent and Le Billon, 2015; Mills, 2017). Recent literature on the shifting political economy of extractive industries in the post-land claim era suggests that IBAs have been used as a tool for corporations to obtain the formal consent of Indigenous communities thus limiting uncertainties and potential project interruptions/disruptions and enhancing the corporations' social license to operate (Cameron and Levitan, 2014; Papillon and Rodon, 2017). However, as Cameron and Levitan (2014) point out, IBAs also naturalize market-based solutions to social, political, and economic problems by providing private capital for services that are (or should be) provided by the state, and by limiting other political avenues available to community members to resist or oppose extractive projects. This last point is particularly important given that the goals and motivations of IBA negotiators –often consultants, lawyers, and land claim executives –may not co-inside with those of the Indigenous community at large. Discrepancies between wide-spread community consent and input and consent obtained through corporate agreements can be exacerbated because the community is often not informed of the content of the IBA until after an agreement is reached (Kulchyski and Bernauer, 2014; Dokis, 2015; Papillon and Rodon, 2017). Impact Benefits Agreements are often signed after preliminary exploratory work and plans have been already been drafted by proponents and can be seen by community governance bodies as their best shot at ensuring benefits for their people in the face of a project that seems inevitable (Bielawski, 2003; Caine and Krogman, 2010, see also Bernauer, 2020). At the environmental review stage, signed Impact Benefits Agreements are often misrepresented by proponents as demonstrating wide-spread community consent for a project (Dokis, 2015). Given the unequal power arrangements between proponents and Indigenous communities in the context of extractive industries, Caine and Krogman (2010, p. 78) argue that Impact and Benefits Agreements “continue to play a pivotal role in community complicity to large scale landscape alternations”.

## 3. Environmental assessment, extractivism, and new technologies of dispossession

Even as environmental assessments recognize harm stemming from extractive industries, they do so in ways that circumscribe how they can register and reveal particular kinds of logics that serve to justify ongoing extraction and consequent dispossession. Critical environmental assessment literature has described how scientific and technical representations of the impacts of extractive industries have been privileged over other ways of knowing and experiencing the world in ways that have helped to elicit support for resource extraction (Dokis, 2015; Bernauer, 2020; Collard et al., 2020). Critiques of environmental impact assessment reflect wider problems associated with Indigenous peoples' participation in state formulated environmental management regimes including the (mis)integration of Indigenous and scientific knowledges in environmental management (Nadasdy, 1999), the use of traditional land use studies and mapping (Thom, 2009; Nadasdy, 2012; Joly et al., 2018), and the tendency for consultation and other participatory practices to reproduce and further entrench the colonial relationship between Indigenous peoples and the state (Nadasdy, 2003; Coulthard, 2014; Dokis, 2015).

Connections between extraction, settler colonialism, and the dispossession of Indigenous peoples have been well documented in the literature. In their reflection on the relationships between extractivsm, settler-colonialism, and nation-building as Canada marked its 150th year, Peyton and Keeling (2017) draw attention to the ways in which the erasure of Indigenous territorialities was achieved through the abstract representation of national extractive spaces, facilitated by technologies of mapping, surveys, and property law. Recent work has shown how new technologies of dispossession such as land claims, resource-co-management, consultation, negotiation, accommodation, and politics of recognition continue to reproduce colonial relations of power, as they work to justify and expand extractive processes (Alfred, 2009; Irlbacher-Fox, 2009; Alfred and Corntassel, 2011; Coulthard, 2014; Simpson, 2017). These new technologies of dispossession are important insofar as they point to a continuing material dispossession alongside the expansion of extractivist frontiers, but also because they erase and dispossess Indigenous intelligences and ways of knowing and being in the world in ways that undercut Indigenous peoples' existence as Indigenous peoples (Simpson, 2017; TallBear, 2019).

Anna Willow describes extractivism as both a material practice and an ideology. Willow (2016, p. 2) writes, “more than just a way of using the land, extractivism is also a way of thinking. It is a way of being in the world”. Characteristics of extractivist thinking and relating include not only the physical rearrangement of landscapes in the pursuit of endless extraction, but also the pervasive conceptualization of “natural resources” as commodities that are used to produce profits well beyond what is necessary for subsistence needs. In *The Great Transformation*, Polanyi (1944) argues that one of the central moves in the transition to market capitalism was the absorption of vast domains of social life under the control of a self-regulating market. As a consequence, not only are productive practices disembedded from general social relations, but all elements of production, including land and labor, or what Polyani calls “fictitious commodities,” must become remade into objects that have a price and that interact with supply and demand. As a consequence, the embodied, sensual, mutually constituted presences found in relational ecologies are rescripted as property relations (see TallBear, 2019). Extractivism not only removes “resources” from their points of origin, but, as Leanne Betasamoksake Simpson argues, “also removes all of the relationships that give whatever is being extracted meaning” (quoted in Klein, 2013). Thus, extractivism's harm is wrought not only on land in a material sense, but also on the webs of relations with whom Indigenous peoples are co-constituted.

Extractivism's reordering of relational ecologies as property relations reflects a common trope in settler colonial thought and practice, where some beings (including humans) are seen as more significant than others. Asch (2014), for example, shows how 19th century theories of civilization have served to justify the position that the principle of temporal priority does not apply with respect to Indigenous peoples because they were considered “too uncivilized” to make proper use of the land and its resources. These theories of civilization persist into the present as evidenced by tests for Aboriginal rights and title in Canadian legal jurisprudence, and the continuing insistence that Indigenous self-determination be reconciled with the underlying sovereignty of the Crown (rather than the other way around). These hierarchies of life extend to the physical environment as well. Kim Tallbear, drawing on the work of Mel Chen, points to the ways in which Eurocentric binaries of life/non-life and culture/nature may be better reflected as gradients of aliveness that reflect a hierarchy of animacy that privileges some forms of life over others. TallBear writes:

“*Dominant cultural ideas point to more gradation are greater and lesser relative degrees of sentience, aliveness, (self)awareness, and agency among different entities…This hierarchy is actualized through the associated verbs/adjectives ‘animate' and ‘de-animate' that refer to the greater and lesser aliveness attributed to humans over other-than-humans, to animals over plants, etc. The animacy hierarchy also de-animates many humans including Indigenous and Black people, by placing them below the Western and often male subject” (TallBear*, *2019**, p. 25)*.

The perception that some forms of life are more valuable than others, and the associated commodity-oriented view of the land as a natural resource to be exploited for profit, contrasts sharply with how many Indigenous theorists conceptualize their relationships with the world. Indigenous scholars from diverse Indigenous traditions have described what Kim TallBear has called caretaking relations as foundational to Indigenous ethical frameworks. These caretaking relations are kin-centric and include obligations across generations of both human and other-than human beings with whom we are all connected: all my relations. Simpson (2017, p. 3) describes these relationships as ecologies of intimacy, which are “designed to generate life—not just human life but the life of all living things”. Dene scholar Coulthard (2014) argues that Indigenous critiques of extractivism are rooted not only in concerns about the dispossession of land, but also in concerns about the dispossession of these relationalities that form the foundations of decolonial Indigenous thought and practice.

Fundamental to these relational philosophies and ethics of care are responsibility, balance, and proportionality, whereby humans should live in such a way as not to interfere with another beings' ability to live well upon the Earth. This is why extractivism, with a focus on continual expansion, can be so problematic. Alfred (2009) explains,

“*Non-Indigenous people may suspect that traditionalist Natives would oppose the types of uses and activities promoted by the state in their nations territories. In fact, this is not the case. Most Native people do not reject modernization or participation in larger economies. However, traditionalists recognize a responsibility to participate in the economy with the intent of ensuring the long-term health and stability of people and the land; in this context, development for development's sake, consumerism, and unrestrained growth are not justifiable” (p. 85*).

Extractivism does impede the ability of all forms of life to live well upon the Earth. Tully describes it as a viscous system, one in which continual extraction, a necessary condition for the survival of the system, undermines life-sustaining webs of human and ecological relationships and gives rise to “the well-known forms of social suffering of modern life, alienation and anomie, the horrendous inequalities in life chances, and the planet of slums and gated communities in which we find ourselves” (Tully, 2018, p. 106). Westman (2013b), as well, points to how extreme extraction in the Alberta oil sands can be interpreted as analogous to a windigo—a cannibalistic monster whose greed and loss of sense of appropriate relatedness to others threatens to consume us all. That extracvitism impedes life-sustaining relations is obvious in scenarios of extreme extraction, such as the oil sands described by Westman, but it is also present and perpetuated, though perhaps more subtly visible, in the extractivist logics employed in what Sax and Tubb (2021) have called the “buzz phase” of development, those processes and practices that include securing investment, drawing up plans, engaging in consultations, and of course in the environmental assessment of extractive projects. Environmental impact assessments, in particular, highlight the stubbornness of an extractivist lens, even in instances when Indigenous peoples are included in these processes on regulatory boards or as publics, precisely because they misconstrue the significance of the impacts of extractivism as a material and an ontological/axiological system.

## 4. Characterizing harm through an extractivist lens: The Mackenzie Gas Project environmental assessment

In 2003, a consortium of five multi-national energy corporations submitted an application to Canada's National Energy Board to build a 1,220 km natural gas pipeline through the Mackenzie Valley. The project sought to develop three natural gas fields in the Beaufort Delta, and to transport natural gas and natural gas liquids extracted from the Northwest Territories to existing pipeline infrastructure in Northern Alberta, and on to southern consumers. At the time that regulatory applications were submitted, the Mackenzie Gas Project was the largest infrastructural project ever proposed in the Canadian north. A significant portion of the pipeline route would be constructed in areas that have no all-weather roads and substantial infrastructure would have been required both for the construction of the pipeline, and for building work camps for laborers, some of which would house up to 400 workers at a time. To provide perspective, one of the communities that I was living in during the regulatory process, Tulit'a, had a population of ~550 residents, and there was a 400-person work-camp that was proposed for 4 km outside of town.

In contrast to scenarios of extreme extraction such as the Alberta oil sands, where the mitigation of harm tends to center around technological remediation of obvious ecological destruction, the immediate ecological footprint of the Mackenzie gas pipeline and related infrastructure was presented by proponents as negligible; a small pipeline right-of-way, and short-term disturbances to the surrounding environment resulting from construction activities. Nonetheless, the construction of a pipeline through the Mackenzie Valley was widely anticipated to open-up a “new energy frontier” in a region with high hydrocarbon potential, but minimal oil and gas or transportation infrastructure, and with a predominately Indigenous population who continue to rely on land-based activities for a significant portion of their food as well as for cultural and spiritual purposes. In this sense, then, the construction of a pipeline through the Mackenzie Valley would most certainly result in transformations of Dene social, cultural, and economic life that extend well beyond divergent forms of land use and economies to include transformations in the caretaking relations that Dene people have with their land. Disruptions to these caretaking relations would be significant, because they form a central part of how Dene people see themselves in relation to the world. Indeed, as Dene scholar Glen Coulthard points out, “the question of land [is] a struggle not only for land in the material sense, but also deeply informed by what the land, as a system of reciprocal relations and obligations can teach us about living our lives in relation to one another and the natural world in nondominating and nonexploitive terms”. Coulthard calls these “place-based foundations of Indigenous decolonial thought and practice” grounded normativity (Coulthard, 2014, p. 13).

A renewed interest in the sociomaterial and political lives of infrastructure has called attention to the ways in which pipelines and other extractive infrastructure assemble diverse and often contested spatial, ideological, economic and political projects (Appel et al., 2018; Spice, 2018). The Mackenzie Gas Project was no exception; for hydrocarbon producers and governments, the pipeline symbolized the promise of the expansion of hydrocarbon frontiers into the Canadian north and the advent of modernity for these northern remote regions. For environmentalists, expanded hydrocarbon infrastructure into the Northwest Territories represented the demise of imagined, pristine, wildplaces. And for Dene people, opening up a hydrocarbon frontier in their homeland had the very real potential to fundamentally upend their way of life. The Mackenzie Gas Project environmental assessment process brought together these various publics with the insistence that all of these myriads of perspectives, and others as well, would be considered in the assessment of such a transformative project.

The regulatory process for the Mackenzie Gas Project was complex given that the pipeline and associated permits fell under cross-jurisdictional authorities. In 2002 a *Cooperation Plan* was established that outlined how the regulatory review would be coordinated. A seven-member Joint Review Pane (JRP) was established to consider potential environmental and socio-economic impacts of the proposed pipeline. The JRP membership included three appointees from the Mackenzie Valley Environmental Impact Review Board, and four from the federal Minister of the Environment (two of which were to be nominated by the Inuvialuit Game Council). At the same time, a National Energy Board (NEB) was established to consider the financial and technical aspects associated with the proposed pipeline including the economic feasibility of the project and project engineering. The NEB received and considered the JRP report and ultimately determined that the pipeline and associated infrastructure was in the public interest and should be built.

The *Terms of Reference for the Environmental Impact Statement for the Mackenzie Gas Project* stated,

“*public participation is an important and integral component of the Environmental Impact Review process, and the Joint Review Panel process will be conducted in a manner that promotes public participation. As described in the Agreement, the Panel will provide opportunities for the public to comment on the adequacy and content of the EIS and also to provide information with respect to potential impacts of the Project” (Inuvialuit Game Council et al.*, *2004**, p. 2)*.

In many ways, the impact assessment process for the Mackenzie Gas Project was exceptional, not just in its complexity and scale, but because it was the first real test of the new regulatory regime.

The *Terms of Reference for the Environmental Impact Statement for the Mackenzie Gas Project* outlined the scope and requirements for the proponents' preparation of the Environmental Impact Statement, including a requirement for the “use and respect for traditional knowledge” (Inuvialuit Game Council et al., 2004, p. 3). Imperial Oil, on behalf of the proponents, submitted a massive eight-volume Environmental Impact Statement (EIS) on 7 October 2004, which outlined their assessment of how the project might impact the environment and communities in the region. Proponents indicated that they had engaged various stakeholders, including potentially affected Indigenous communities to inform the preparation of the EIS, by conducting interviews, group meetings, by holding community dinners, open houses, and workshops. Information gleaned from these encounters were said to have informed field studies and assisted in identifying impacts to the land and people. The proponents also retained a group of consulting companies including AMEC Earth and Environmental Limited, IMG Golder Corporation, KAVIK-AXYS Inc. and TERA Environmental Consultants to complete environmental field studies, biophysical and socio-economic evaluations and assessments and to provide support to the proponents through the regulatory process. The EIS makes clear that “the consultants are actively involved in the project design providing input to the engineers, planners and management. The team used the extensive skills and experience of its assessment members to design and complete the required biophysical and socioeconomic studies and consultations to evaluate the probable effects of the proposed development and operation of the project” (MGP., 2004a, p. 1–30).

A number of studies have made the point that environmental and social impact assessments are conducted in ways that privilege Western knowledge systems and are not consistent with sound contemporary anthropological methodological or scholarly practices (Povinelli, 1995; Natcher, 2001; Westman, 2013a; Wyndham, 2017; Baker and Westman, 2018). For example, an analysis of the environmental impact statement for the Mackenzie Gas Project shows no evidence of engagement with literature by Indigenous scholars on the colonial nature of research paradigms and how these have alternatively distorted and neglected Indigenous methodologies and ways of knowing^1^ (Smith, 1999; Battiste and Henderson, 2000; Wilson, 2001). Conducted in such a way, environmental impact statements tend to neglect the affective ontological and relational significance of the impacts of extractive industries as perceived by Indigenous peoples, while substituting the promise of an optimistic future based on increased employment opportunities, resource royalties, and involvement in capitalist systems of production and consumption. Though proponents of the Mackenzie Gas Project indicated that they had engaged affected Indigenous communities in the preparation of their environmental impact statement, and that information gleaned from these encounters informed field studies and assisted in identifying potential impacts to the land and people, a review of the Environmental Impact Statements findings suggests that Dene axiologies and ontologies, as I understand them, were not well represented. For example, the animacy hierarchy, described by Tallbear, was built into the methodological structure of the environmental impact statement, as some species of plants and animals were included for assessment, while others were not. The Mackenzie Gas Project environmental impact statement, for example, only assessed the impacts of the proposed pipeline on wildlife species identified as ‘valued ecosystem components,' such as barren-ground caribou, grizzly bear, woodland caribou, moose, marten, lynx, beaver, amphibians, greater white-fronted goose, snow goose, tundra swan, scaup, peregrine falcon, whimbrel, lesser yellowlegs, arctic tern, boreal chickadee, and marine mammals. According to the environmental impact statement, these species were selected “from a list of candidate species that either had regulatory status designation, economic or public profile value to northern communities, a particular ecological importance, or a combination of these” (MGP., 2004a, p. 10–12). Other species, such as mice or rock ptarmigan were excluded from assessment altogether. The disarticulated and selective assessment of the impacts of project activities on some species, but not others, conceals and confuses the caretaking relations that form the basis for Dene place-based grounded normativity, and goes against Dene ethical frameworks that insist on the ability for all beings to live well together upon the Earth. While I was living in the Sahtu, Dene people pointed out their concerns about the impacts of the pipeline on a number of different beings not included in the environmental impact assessment. One Dene grandfather and hunter said during one of our conversations,

*A couple of years ago, they did some exploring right across from here. When they were drilling, we could feel the ground shake. The caribou could feel it. They went in a different direction, so you can pretty well see how animals react to the exploration. Things are going to be not as quiet when the pipeline goes through. We know how much damage it is going to do. All of the water, animals, even the mouse—all of the mouse dens, even beaver. They don't see that. But we the people who live off of the land, we see it*.^2^

In their environmental impact statement, Imperial Oil acknowledged that the pipeline and related infrastructure would have adverse effects on the biophysical environment, but they argued that the project footprint would disturb only a small portion of the Mackenzie Delta and Valley, and in their assessment these effects would be so low as to be insignificant (MGP., 2004b, p. 7). Any disturbances to biophysical components of the environment were interpreted as “not significant” because they covered a small area that could be recovered through remediation of disturbed sights after a short (~30-year) period of time. In fact, Imperial Oil argued that in some instances, such as the availability of moose habitat, landscapes would actually be improved after project activities (MGP., 2004a, p. 5: Section 10, 3).

Given that the pipeline corridor would run through regions with limited exposure to hydrocarbon extraction outside of the Norman Wells Proven Area, a significant portion of the impact assessment was focused on the potential impacts on Indigenous communities as a result of a transition to a hydrocarbon-based economy. Imperial Oil described possible impacts to socio-economic indicators including demographic changes and associated stresses on community services such as housing, health, and transportation infrastructure, increased costs of living, and the need for education and training so that Indigenous people could take up employment in new industries. Yet Imperial Oil characterized the net effect of this transition as positive, highlighting the economic benefits that the pipeline would bring for individual employment, for revenue generated for Indigenous businesses and governments through Access and Benefits Agreements, and for territorial and federal governments in the form of resource royalties. The Aboriginal Pipeline Group (APG), a corporation formed to represent the interests of the Inuvialuit, Gwich'in and Sahtu, became a joint venture partner making regional land claim bodies one-third owners of the pipeline. An ownership stake in the pipeline was seen by Indigenous land claim organizations as an important means to secure long-term benefits from the pipeline. The APG received an $80 million dollar loan from TransCanada Corporation to cover the up-front costs for regulatory reviews and construction in exchange for TransCanada securing a five percent interest in the pipeline. Yet, while the APG would have held an ownership stake in the pipeline proper, it did not own the anchor fields and so it would have to find additional natural gas to ship through “their section” of the pipe. Thus, revenues for the APG would grow only with additional exploration and production of natural gas, and the full APG 33 percent ownership would be realized only if the shipment of gas through the pipeline increased in volume to more than 400 million cubic feet per day. As a consequence, Indigenous ownership in the pipeline would be profitable only if it resulted in the intensification of hydrocarbon extraction, a future scenario that many Indigenous community members said they wanted to avoid. Further, any profits initially generated by the APG from pipeline tolls would be used to repay the loan from TransCanada and would not immediately be transferred to shareholders.

The general presumption within the Environmental Gas Project environmental impact statement was that any negative socio-economic impacts associated with a transition to a hydrocarbon-based economy would be offset by the economic benefits that it would bring. It was recognized that there may be some stress on community infrastructure, but that this would not be significant as mitigation measures would be put in place to keep the work camps largely self-sustaining, and all employees (including Dene employees who live in nearby communities) would be required to stay at the work camps for the duration of their rotational cycle. Socio-economic impacts were characterized as manageable through mitigation measures, often in the form of policies on workplace safety and training, and through education and training initiatives for Indigenous people, including what they called “financial literacy training” that would teach Dene people the principles of financial planning and saving. Financial literacy training was especially distasteful to many of the Dene people that I talked with during my time in the Sahtu because saving money (a practice associated with greed) is antithetical to Dene norms of generosity that form a central component of Dene ethical frameworks, and because it “shows a lack of faith that the land and the Creator will provide what people need.”^3^

Indicators of the anticipated socio-economic impacts of the proposed project were made visible through the quantification of data such as employment rates and household income, amount of capital spending by the project proponents over the four-year construction period and subsequent operations period, government revenue, and demographic changes. However, much trickier to quantify were the impacts of the project on what was defined in the environmental impact statement as “traditional culture.” Accounting for impacts on traditional culture presented a problem: how could the life-sustaining relational ecologies that inform place-based normativity as described by Coulthard (2014) be evaluated and measured? The environmental impact statement did not explicitly define what was meant by “traditional culture,” other than to say,

“*Survival by harvesting food resources nourished by the land is the ethos, the essential center, of Aboriginal cultures. These cultures are sustained today by community influences that communicate preferences and needs, and encourage harvesting of traditional foods. Sustaining the knowledge, lore, and skills necessary for harvesting these foods depends on motivation and the time to engage in these activities” (MGP.*, *2004a**, p. 6–28)*.

Nowhere in the environmental impact statement was there mention of caretaking relations, or of the importance of the relational ecologies that form the foundations of Dene ethical frameworks. Yet, the environmental impact statement did operationalize three key indicators whereby “traditional culture” could be quantified and measured. Involvement in traditional culture was presented in numerical terms: as (1) the percent of adults who participate in traditional harvesting, (2) the amount and market value of country food consumed by residents, and (3) the percentage of people who speak an Indigenous language.

Representing culture quantitatively allowed for the creation of metrics that could be used by Imperial Oil to evaluate the “significance” of the project's impacts on culture. To paraphrase geographer Robertson (2006, p. 369) it is not that culture was excluded in determining the significance of the impact of project activities, but rather how it was *included* that matters. In his work on the development of market-based ecosystem services, Robertson showed that techniques used to measure the value of ecosystem functions in rapid assessment methods often require simplification and categorization so that complex relationships can be made visible in the logics of capital. In his assessment, messy and complex qualities of nature are often translated into commoditized values in order to make them intelligible in commodity markets. For Robertson (2006, p. 382), the difference between evaluating commodities such as a loaf of bread and ecosystem functions are that “capital logics require information about ecosystem services that scientists cannot provide in an uncontroversial way”; that is, determining the “value” and certification of complex relations rests in shaky articulations between science and capital which require the translation of hard-to-quantify properties into logics that make them economically coherent. Fabiana Li notes similar logics of equivalence in her analysis of a large gold mine in Chile, with the result that scientists and engineers not only work to make diverse entities measurable and comparable, but that this fictitious translation allows companies to articulate the environmental effects of a mining project in ways that are commensurate with the mining companies' mitigation plans (Li, 2018).

**Figure 1:**
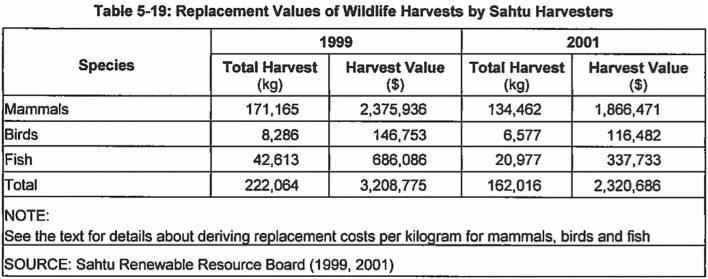
Table of the calculation of the replacement value of wildlife harvests by Sahtu harvesters included in the Mackenzie Gas Project environmental impact statement, prepared by Imperial Oil (MGP., 2004a, Vol. 4, Section 5: 5–19).

The reification of culture into quantifiable indicators in the Mackenzie Gas Project environmental impact statement similarly translated Dene axiologies and ontologies, along with subjective qualities of lived experiences, into numerical representations that could generate capital equivalencies. The environmental impact statement suggested that most of the impacts on traditional culture could be mollified by corporate policies such as flexible work schedules to accommodate hunting and other land-based activities, supporting community events related to language and culture, and through cultural competency training for non-Indigenous workers. But Imperial Oil acknowledged that other effects could simply not be mitigated by programs and policies. This was particularly the case for hunters and trappers who would be affected by the movement of wildlife out of work areas during pipeline construction, and longer-term changes to animal habitat, predation, and migration. Yet because hunting was measured only as an activity that people do (rather than for what it means for Dene hunters and trappers and the animals with whom they are engaged), and the outcomes of these activities were represented as consumable food or as commoditized furs, the effect of these losses was characterized as measurable in economic terms –as an actual or potential loss of revenue in the case of trapping, and as an interruption in the availability or location of wild game for hunters who rely on animals to feed their families (see Figure 1).

As a means of addressing these losses, Imperial Oil offered to compensate hunters and trappers who could demonstrate that their livelihoods would be affected by project activities. The compensation value for furs was based on trappers' records of yields in pervious trapping seasons, along with anticipated future fur prices. Compensation for loss of hunting or fishing relied on similar records, and the value of the loss of country food was calculated based on the cost of replacing the harvested animal with store-bought beef, chicken, or fish. Evaluated in this way, the environmental impact statement neglected the place-based caretaking relations central to Dene ethical frameworks and made entire realms of life that are differently valued, experienced and known by Dene people equivalent to a trip to the grocery store. This quantification subverted localized systems of meaning that are not singly economic in nature, but also characterize how people think about the world and their place in it.

## 5. Maintaining relations (or how a moose is not like money)

When Dene people talk about their relationships with animals, it is clear that they resist the notion that animals are equivalent to capital. Testifying before the Joint Review Panel for the Mackenzie Gas Project, Morris Neyelle, a grandfather and hunter from Déline, explained: “without the animals on the land, as Aboriginal people, it's not worth living. That's how it is… even though you gave us lots of money, but if there's no animal, what's the use?”^4^

Government of the Northwest Territories Bureau of Statistics data indicates that at the time that the pipeline was proposed, more than half of households in the Sahtu communities where I worked relied on country food for the majority of their diet (GNWT, n.d.). Country food is not only preferred by many Dene people, obtaining food from the land contributes to food security in a region where the price index for food in 2004 was 32% higher than food costs even in Inuvik (MGP., 2004a, p. 4: Section 5, 19). Dene people often refer to the land as their “grocery store” and as their “bank” and describe hunting and fishing as far more predictable forms of food provision than purchasing food in the grocery store which, as is the case in many remote Northern communities, can be prone to sharp fluctuations in cost and availability. At least some Dene elders associate store-bought food with disease and describe commercial forms of industrial food production as interfering in an animal's ability to live a good life.^5^

Country food is also implicated in a moral economy where meat and other parts of animals are regularly shared across households and communities and without monetary exchange. During my fieldwork, Morris Neyelle often explained that the exclusion of country food from market exchange was an important part of Dene land-based ethics. “What the land provides is free,” he would say, “and it should be given to others without payment.” Sharing country food not only creates networks of balanced exchange that enhance food sovereignty, it also fosters strong community ties. Asch (1977, p. 19) describes the extended household among Dene people in Wrigley as a means of retaining a (collective) band-oriented form of productive practice that has mediated the “individualized, self-sufficient nuclear orientation” of market-oriented modes of production. In her work on the effects of intensified oil and gas extraction for Cree people in Lubicon Lake, Rosemary Brown identified disruptions to food-sharing networks as a result of a transition to store-bought food because, as she writes, “store bought food could not be used to cement social and ritual ties in the same way that moose and other game meat had” (Brown, 1997, p. 198). Brown notes that disruptions in these networks of balanced exchange had significant political consequences including shifting relations of co-operation and mutual assistance to ones of dependence on the state. Dene people, too, highlight the differences between life in the bush, seen as intimately tied to Dene identity and freedom, and life in the communities, which is often associated with imposed colonial institutions and dependency. As Déline grandmother Carolyn Yukon explained: “when we're all together, when we're all out on the land, we don't think of nothing. We don't think about our jobs. We don't think about our community. We don't think about paying our bills. The scenery is so beautiful out there, and that's why we love our land so much. We don't want anybody to take it away from us.”^6^ Thus, Dene productive activities associated with obtaining material sustenance from the land might more closely resemble what (Hazareesingh and Maat, 2016, p. 6) call anti-commodities, or “local productive practices associated with values other than the purely economic” that not have not only endured but stand in opposition to colonial formations and various modes of commodification inherent in modern capitalism.

While animals are sought for physical sustenance, Dene people value relationships with animals for a variety of reasons that lie outside of economic domains. Ethnographic literature on subarctic Indigenous peoples describes the central location of non-human animals in Indigenous ontologies, cosmologies, and axiologies (Brightman, 1993; Anderson and Nuttall, 2004; Nadasdy, 2007). Animals are seen as relatives, helpers, and teachers (Feit, 2004). For Dene, the assistance of animals is sought for metaphysical purposes including the acquisition of knowledge and power for healing, prophecy, locating (or calling) animals, or for gifts that otherwise might help the people to survive and live a good life (Andrew, 2018). Animals are thought to “have pity” on humans, especially those who behave with humility and who actively seek out these relationships. What much of this makes clear is that animals are not conceptualized as “things” (Feit, 2004), but rather as intentional beings who willfully offer themselves or their knowledge to human persons. These socialities extend beyond animal-human relations to various components of the natural world including wind, water, mountains, and plants.

In order to receive life-sustaining gifts from animals, humans need to maintain proper relationships with them. Dene people talk about the importance of maintaining Dene Law, an ethical framework that contains important instructions that enable human and animal communities to live together on the land (Blondin, 1990). In general, Dene law obliges Dene and animals to help each other, not interfere in each other's ability to live a good life, and to treat one another with kindness. These ethical principles extend to relationships between human persons and communities as well. I remember a conversation with a Dene elder who wondered why non-Indigenous societies are preoccupied with expansion. He said, “Dene and Mola^7^ have two different lifestyles. Mola even go up to the moon and stars. Why are they doing that? Why are they bothering things like that? They should just leave it” (see text footnote ^2^).

When people and animals uphold caretaking relations, and the principles contained within Dene law, all beings are able to live well together on the Earth. When this happens, as Déline Elder Charlie Neyelle explained, “we are very pleased with each other. The animals are very pleased with us and we're very pleased with them because we obey those universal law.”^8^ When humans interfere in an animal's ability to live well on the land –by building a pipeline, for example –humans break the universal law in ways that cannot be mitigated by corporate policy or through technological remediation; nor can these relationships be mended by monetary compensation. It is not that Dene people are opposed to hydrocarbon development, but that it should be done proportionately, and with care, giving weight to the current and future costs for all living beings. As one Dene elder said to me once, “if the world really needed oil we would give it, if it was really scarce maybe then they could work on it. But now they have lots, they don't need it” (see text footnote ^2^). Thus, for Dene people, impacts brought about by extractive industries are significant not only for their “ecological footprint,” but for the disruption to socialities within and between human and other-than-human communities.

## 6. Conclusion

In the final report and recommendations of the Mackenzie Gas Project environmental assessment titled, *Foundation for a Sustainable Northern Future*, the Joint Review Panel recommended that the pipeline be built, subject to a number of recommendations. In their assessment, “the MGP offers a unique opportunity to build a sustainable future in the Mackenzie Valley and Beaufort Delta regions. The project itself, as long-term infrastructure, provides a key basis for future economic development.” The Joint Review Panel concluded that, “the adverse impacts of the Mackenzie Gas Project and the Northwest Alberta Facilities would not likely be significant and that the Project and those Facilities would likely make a positive contribution toward sustainability” (JRP, 2009, p. v). The kind of language used in the Joint Review Panel recommendation reflects what Rosemary- Collard and Dempsey (2022) identify as a temporal strategy often employed by settler colonial states to mediate the contradictory role of the state as committed to reconciliation and environmental protection on the one hand, and economic growth on the other. These temporal fixes consistently invoke an idealized and different kind of future, while simultaneously justifying historical and ongoing environmental degradation and further entrenching extractive material and ideological practices.

Ultimately, the pipeline was never built. Rising costs for construction (to more than $16 billion CND), coupled with a decline in natural gas prices as a result of an increase in supply in more accessible regions in the United States, made the project economically unprofitable and so the consortium of energy companies abandoned the project in 2017. Pipeline proponents also blamed the lengthy environmental assessment and regulatory process as a contributing factor to the project's demise. Speaking to the CBC, a spokesperson for Imperial oil said that they did not anticipate the length of time that it would take for the project to receive approval: “Our initial estimate for the timing for the regulatory process was somewhere between 22 and 24 months. We filed for regulatory approval in 2004 and we received final regulatory approval in 2011. I'll leave it up to you to decide if that is a reasonable amount of time for a significant capital investment project” (as quoted in Strong, 2017). Still, the decisions to both approve and consequently abandon the pipeline were economic ones, driven not by environmental or sociocultural reasons, but by the quest to expand into new extractivist frontiers, and to maximize profits and minimize losses. As Coulthard rightly notes, “unlike the discourse of sustainability underwriting Dene claims, which sought to establish political and economic relations that would foster the reciprocal wellbeing of the people, communities and land over time, sustainability now refers primarily to the economic sustainability of capital accumulation itself” (Coulthard, 2014, p. 77).

The inclusion of various publics in the Mackenzie Gas Project environmental assessment process, at least theoretically, held the promise that Indigenous peoples' voices and concerns would be represented in the decision about the pipeline. Yet, the persistent foundation of extractivist logics that underpin environmental assessments such as the one conducted for the Mackenzie Gas Project serve as an important reminder that the creation of co-management and other “inclusive” institutions and participatory processes alone are insufficient to meet UN Sustainable Development Goal #16. That is, inclusive institutions cannot be created by simply inserting diverse publics into pre-existing systems of authority without simultaneously disrupting and dismantling the systems of coloniality, power, and inequity upon which they are based.

As this paper shows, the mechanisms and extractivist logics used by proponents in their environmental impact statement made the anticipated impacts of the pipeline and associated infrastructure visible only in particular kinds of ways: as quantitative indicators that distorted and obscured the impacts of extractive industries on caretaking relations as described by Indigenous peoples. As a consequence, the significance of impacts associated with extractive industries were not only minimized but were also mischaracterized as ones that can be remedied with economic solutions. As such, these techniques simultaneously work as contemporary technologies of colonialty and dispossession as relational ecologies and place-based ethical engagements were made invisible through the violence of numbers.

Recent scholarship has called attention to and cautioned against these forms of misrecognition, especially as emergent discourses of reconciliation and recognition threaten to include Indigenous peoples and knowledges in the Canadian political landscape only in symbolic rather than substantive ways (Irlbacher-Fox, 2009; Coulthard, 2014; Tully, 2018). As Leanne Betsamosake Simpson writes,

*I think the insight that settler colonialism is formed and maintained by a series of processes is important because it recognizes that the state sets up different controlled points of interaction through its practices –consultations, negotiations, high-level meetings, inquiries, royal commissions, policy and law for instance, that slightly shift, at least temporarily and on microscales, our experience of settler colonialism as a structure…It can appear or feel as if the state is operating differently because it is offering a slightly different process to Indigenous peoples….Colonialism as a structure is not changing. It is shifting to further consolidate its power, to neutralize our resistance, to ultimately fuel extractivism” (Simpson*, *2017**, p. 45–46)*.

Simpson, and others, offer a cautionary tale where processes of inclusion give the illusion of conciliatory relationships, but are only permitted when they do not fundamentally threaten the settler state and the associated economic practices on which it depends. Simpson (2017, p. 50) makes the point that the inclusion of Indigenous culture in the Canadian political and social landscape can be done in ways that don't fundamentally alter the colonial relationship; she writes, “Language, cultural expression, and even spirituality don't (necessarily) pose an unmanageable threat to settler colonialism because cultural resurgence can rather effortlessly be co-opted by liberal society”. However, these forms of accommodation and recognition leave the fundamental structure of settler colonialism in place. Unless accompanied by corresponding political reformations that include dismantling systems of colonialism, politics of accommodation and recognition offer little hope of genuine reconciliation.

Philosopher James Tully has pointed out that reconciliation between Indigenous and non-Indigenous peoples will not succeed without the reconciliation of all human beings, Indigenous and non-Indigenous people alike, with the living earth (Tully, 2018, p. 84). Part of reconciliation with the living earth requires a close examination of the logics of extractivism including an orientation to the limitless use of “natural resources” for profit at the expense of other living beings and the transformation of life-sustaining relations into relations of property. The stakes are high. As Tully notes, “if reconciliation fails, the crisis-ridden system crosses a tipping-point and collapses in whole or part, taking many of the forms of life with it” (Tully, 2018, p. 95). In that sense, taking caretaking relations seriously may be instructive, not just for the survival of Indigenous peoples, but for the survival of us all.

## Data availability statement

Fieldnotes and interview transcripts are restricted to protect the confidentiality of research participants. Requests to access the datasets should be directed to carlyd@nipissingu.ca.

## Ethics statement

The studies involving human participants were reviewed and approved by University of Alberta Research Ethics Board. The patients/participants provided their written informed consent to participate in this study. Written informed consent was obtained from the individual(s) for the publication of any potentially identifiable images or data included in this article.

## Author contributions

The author confirms being the sole contributor of this work and has approved it for publication.

## Footnotes

^1^The references included are only those that were published before the Mackenzie Gas Project Environmental Impact Statement was prepared. Since then, a number of additional influential manuscripts have been published by Indigenous authors on the importance of Indigenous research methodologies including those by Longboat (2008), Wilson (2008), Kovach (2009), and Davidson and Davidson (2018) among others.

^2^Fieldnotes, 12 September 2006.

^3^Fieldnotes, 3 September 2006.

^4^Morris Neyelle, JRP Hearing Transcripts, 3 April 2006, vol. 16, 1635.

^5^John Gully, JRP Hearing Transcripts, 10 April 2006, vol. 20, 1997.

^6^Carolyn Yukon, Joint Review Panel Hearing Transcripts, 3 April 2006, vol. 16, 1639.

^7^Mola is a slavey term used to refer to persons of European descent. The term refers to a person with a ringed finger, and was first adopted in reference to fur traders who wore wedding rings.

^8^Charlie Neyelle, Joint Review Panel Hearing Transcripts, 3 April 2006, vol. 16, 1644.
